# Chronic Administration of Benzo(a)pyrene Induces Memory Impairment and Anxiety-Like Behavior and Increases of NR2B DNA Methylation

**DOI:** 10.1371/journal.pone.0149574

**Published:** 2016-02-22

**Authors:** Wenping Zhang, Fengjie Tian, Jinping Zheng, Senlin Li, Mei Qiang

**Affiliations:** 1 Department of Neurotoxicology, School of Public Health, Shanxi Medical University, Shanxi, Taiyuan, 030001, China; 2 Department of Children and Adolescences, School of Public Health, Shanxi Medical University, Shanxi, Taiyuan, 030001, China; 3 Department of Medicine, The University of Texas Health Science Center at San Antonio, San Antonio, TX, 78229, United States of America; University of Texas Health Science Center San Antonio Texas, UNITED STATES

## Abstract

**Background:**

Recently, an increasing number of human and animal studies have reported that exposure to benzo(a)pyrene (BaP) induces neurological abnormalities and is also associated with adverse effects, such as tumor formation, immunosuppression, teratogenicity, and hormonal disorders. However, the exact mechanisms underlying BaP-induced impairment of neurological function remain unclear. The aim of this study was to examine the regulating mechanisms underlying the impact of chronic BaP exposure on neurobehavioral performance.

**Methods:**

C57BL mice received either BaP in different doses (1.0, 2.5, 6.25 mg/kg) or olive oil twice a week for 90 days. Memory and emotional behaviors were evaluated using Y-maze and open-field tests, respectively. Furthermore, levels of mRNA expression were measured by using qPCR, and DNA methylation of NMDA receptor 2B subunit (NR2B) was examined using bisulfate pyrosequencing in the prefrontal cortex and hippocampus.

**Results:**

Compared to controls, mice that received BaP (2.5, 6.25 mg/kg) showed deficits in short-term memory and an anxiety-like behavior. These behavioral alterations were associated with a down-regulation of the NR2B gene and a concomitant increase in the level of DNA methylation in the NR2B promoter in the two brain regions.

**Conclusions:**

Chronic BaP exposure induces an increase in DNA methylation in the NR2B gene promoter and down-regulates NR2B expression, which may contribute to its neurotoxic effects on behavioral performance. The results suggest that NR2B vulnerability represents a target for environmental toxicants in the brain.

## Introduction

It is well known that humans uptake polycyclic aromatic hydrocarbons (PAHs) in several ways during daily environmental exposure [1]. Benzo(a)pyrene (BaP), a high molecular weight PAH with a five-ring polycyclic aromatic hydrocarbon, is one of the most studied members of PAHs. As one of the well-recognized environmental pollutants, BaP can be derived from many sources, such as coal-processing waste products, petroleum sludge, asphalt, creosote and tobacco smoke, as well as byproducts of indoor activities, such as cooking oil fumes. All of these sources can produce high levels of BaP [2]. Due to its cytotoxic, mutagenic, genotoxic and carcinogenic properties, exposure to BaP is associated with many adverse biological effects, including immunosuppression, tumor formation, teratogenicity, and hormonal disorders [3–6]. Compared to the carcinogenicity of BaP, the neurotoxic effects have received less attention. As a lipophilic compound, BaP easily crosses the blood-brain barrier, giving it direct access to the central nervous system (CNS). Moreover, when exposed to high levels, BaP and its metabolites tend to accumulate both in the blood and in the brain [7], thus causing neurological impairments [8, 9]. BaP-induced neurotoxicological effects have been demonstrated in both adult and developing brains, particularly with regard to deficits in long-term spatial memory due to its effects in the hippocampus and prefrontal cortex (PFC) [10–12]. Evidence from human epidemiological studies has also shown that unintended prenatal exposure to BaP adversely affects fetal development, resulting in low birth weight and reduced head circumference, as well as neurobehavioral damage in the offspring [13].

Studies have recently been published investigating the mechanisms underlying BaP’s effect on the CNS. Chronic exposure to BaP has been shown to modulate the levels of several neurotransmitters, including noradrenalin, acetylcholine, dopamine and serotonin [14]. The N-methyl-D-aspartate receptors (NMDARs) are a class of ionotropic glutamate receptors that are essential for neuronal development, synaptic plasticity, cognitive function and cell survival. The level of NMDARs at synapse critically regulates brain function and cell survival. A functional NMDA channel consists of four or five subunits from two sequence-related subunit families, namely NR1 and NR2A-D subunits [15, 16]. Increasing evidence is demonstrating the importance of the NR2B subunit in determining the pharmacological and functional properties of the NMDA receptor, particularly in working memory [17–19]. Transgenic mice with overexpression of NR2B in the forebrain exhibit superior learning and memory abilities when engaged in various behavioral tasks [17, 20]. In agreement with this observation, NR2B knockout mice performed worse on the Morris water maze task and contextual fear-conditioning [21, 22]. Additionally, patients who have died of Alzheimer’s disease displayed cognitive deficits as the most common symptoms with significant reductions in NR2B expression in the hippocampus and cortex [23, 24]. Importantly, studies have shown that the NMDARs are sensitive to various micro-pollutants including PAHs, tetrachlorodibenzo-para-dioxin, polychlorinated biphenyl, lead, methylmercury and toluene [12, 25–28]. Gestational exposure to a mixture of environmental pollutants, including BaP induced a long-term deficit in learning ability and long-term potentiation (LTP). These deficiencies are associated with a decrease in NR2B gene expression in the hippocampus [29]. Recovery of NR2B expression has a protective effect in developing rats with BaP-induced learning impairments [30]. In addition, prenatal exposure to BaP impaired cortical neuronal function during later-life and diminished mRNA expression of NR2B in the offspring [31]. However, the exact mechanisms mediating the neurotoxicity resulting from exposure to BaPs remain unclear.

Epigenetic mechanisms, including modulation of chromatin structure and DNA methylation, have been shown to be potent regulators of gene transcription in the CNS. Experimental and epidemiological studies have linked environmental factors to aberrant changes in epigenetic pathways [32]. In our previous research, we have identified the role of DNA methylation in the expression of the NR2B associated with the effect of ethanol on neuroadaptation [33, 34], suggesting that NR2B is a target for environmental insults. Therefore, in the present study, male mice were chronically exposed to BaP to test the hypothesis that the changes in DNA methylation in the NR2B promoter mediate BaP-induced abnormalities.

## Materials and Methods

### Animals

Eight-week-old male C57BL/6J mice were used in this study. The animals were obtained from the research animal center of Shanxi Medical University (Taiyuan, China) for Y-maze test and Jackson Laboratory (Bar Harbor, ME, USA) for all rest experiments. All animals were given a minimum of 14 days acclimation period prior to initiation of any experiments, and were housed on a 12/12-h light/dark cycle chambers and fed *ad libitum*. All animal procedures were approved by the Animal Care and Use Committee of Shanxi Medical University and the Institutional Animal Care in China and Use Committee of the University of Texas Health Science Center at San Antonio in US. Since a Y-maze test was conducted in Shanxi Medical University in China, which resulted in an obvious trend of lower percentage of spontaneous alternations compared to the control mice, the same procedures were used in the University of Texas Health Science Center at San Antonio in USA for the BaP treatment and Y-maze experiments. The results of Y-maze test shown no difference compared with those in China (data not shown). Therefore, we continued to conduct open field test and the tissues were isolated from those animals for qPCR and DNA methylation analysis.

### BaP treatment

BaP was purchased from Sigma (A2385, St. Louis, MO) and dissolved in olive oil. Animals were randomly divided into 4 groups, namely, control (CTL), olive oil only; BaP1.0, 1.0 mg/kg; BaP2.5, 2.5 mg/kg; and BaP6.25, 6.25 mg/kg. The mice were administered with different doses of BaP by intraperitoneal [35] injections (volume 50 μl) twice a week for 12 weeks.

### Y-maze tests

**Fig 1:**
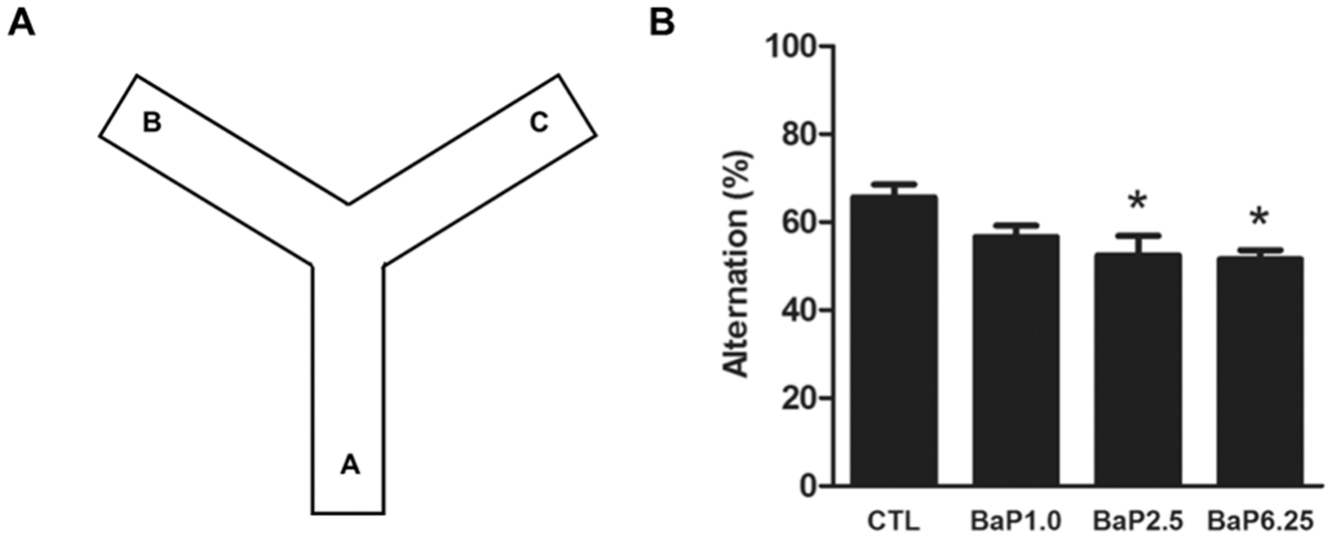
BaP impaired short-time memory in the Y-maze test. (A) A schematic view of Y-maze with three arms, i.e., A, B, and C. (B) Y-maze test was used to evaluate short-term memory of the animals after BaP chronic exposure. Percentages of spontaneous alternation (%) were calculated and the values are presented as the mean ± SEM. *, *p* < 0.05 compared to CTL.

Spontaneous alternation behavior in Y-maze has been used to assess short-term spatial memory. The Y-shaped maze used in this study is a three-arm horizontal maze with 120° angles between each arm measuring 40 x 5 x 15 cm symmetrically. The apparatus was placed on the floor of the experimental room and was illuminated with a 100-W bulb 200 cm above the floor. Mice were initially placed at the end of one arm (A) (see Fig 1A) and were allowed to freely explore the three arms. The number of arm entries and the number of triads were recorded to calculate the percentage of alternation. Over the course of multiple arm entries, the entry sequence (e.g., ABC, BCA or CAB, where letters indicate code of arms) was recorded manually over 6 min periods. Because animals have a tendency to enter a less recently visited arm, spontaneous alternation was determined for successive entries into the three different arms in overlapping triplet sets. An actual alternation was defined as entries into all three arms consecutively, i.e., ABC, CAB, or BCA but not BAB. An entry was defined as placing all four paws within the boundaries of the arm. The percentage of alternation (% alternation) was calculated as spontaneous alternation/ (total number of arm entries—2) x100.

### Open field tests

Mice were placed at a corner of the open field chamber that consisted of a 40 cm (W) × 40 cm (L) × 30 cm (H) acrylic box with opaque walls [36]. They were allowed to freely explore the whole field for 5 min of recording. The floor was divided into 16 equally-sized squares. The central area was defined as the 20 × 20 cm interior central portion of the chamber, while the duration in the periphery zone within 5 cm against the chamber wall was recorded as an index for assessing anxiety-like tendency in mice. A camera was mounted above the open box to record locomotor activity. The behavioral measurements analyzed included the total running grids, the latency and times of entering the center of the area, and the total time in the periphery zone. The chamber was cleaned with dilute alcohol (20% v/v) and dried between animals.

### RNA isolation

Mice were cervical dislocated and sacrificed by decapitation. The brains were quickly removed. The tissues from the PFC and hippocampus were dissected, and then snapped in liquid nitrogen before storage in -80°C. Total mRNA was extracted from these tissues using Agilent Total RNA Isolation Mini kit (Agilent Technologies, Inc., Cedar Creek, TX, USA) according to the manufacturer’s instructions. RNA concentration and integrity were determined by using a NanoDrop1000 (Thermo Scientific Inc., Waltham, MA, USA).

### Quantitative PCR (qPCR) assay

NR2B mRNA expression was measured by qPCR. Two-step qPCR was performed as previously described [37]. Briefly, using 1 μg of total RNA as template, single-stranded cDNAs were synthesized using random hexamers and the TaqMan Reverse Transcription Reagent Kit (Applied Biosystems, Branchburg). qPCR was performed using the ABI 7900 sequence detection system. The 2^−ΔΔ^Ct method was used for quantification with 18S as the endogenous control.

### Genomic DNA extraction

To determine NR2B gene DNA methylation status, genomic DNA from the PFC or hippocampus was isolated as previously described [33]. Briefly, DNA was extracted with ChargeSwich gDNA Mini Tissue kit (Invitrogen, Carlsbad, CA, USA) according to manufacturer’s protocol. DNA concentration was quantitated with Nanodrop 1000 (Thermo Scientific, Waltham, MA, USA).

### DNA methylation analysis

DNA methylation analysis has been described in a previous study [33]. Briefly, genomic DNA (1 μg) isolated from the PFC or hippocampus using Blood and Cell Culture DNA kit (Qiagen), was treated with bisulfate using Zymo DNA Methylation Kit (Zymo research, Orange). Bisulfate-treated DNA was eluted in 10 μl volume, and 1 μl was used for each PCR reaction. PCR was performed with primers biotinylated to convert the PCR product to single-stranded DNA templates. The PCR products (10 μl) were then sequenced by pyrosequencing PSQ96 HS System (Biotage, Kungsgatan, Sweden) following the manufacturer’s instructions. The methylation status of each locus was analyzed individually as a T/C SNP using QCpG software.

### Statistical analysis

The results were analyzed with GraphPad Prism 5.0 (GraphPad Software, La Jolla, CA) and presented as the mean ± SEM. Significant differences between treatment groups and the control were determined using one-way ANOVA followed by Neumann—Keulls *post hoc test* (*p* < 0.05) or student *t-test* (p < 0.05). For qPCR, significant differences between treatments were determined on the linearized 2^−ΔCT^ values.

## Results

### BaP impaired short-time memory

Twenty seven mice received BaP in three different doses groups: 1.0, 2.5 and 6.25 mg/kg (n = 9 per group) for three months. The Y-maze test was used to assess the effects of BaP on the short-term memory of these mice. The results from one-way ANOVA analysis showed a significant effect of BaP treatment on short-time memory (F_(3, 32)_ = 3.361, *p* < 0.05) (Fig 1B). Mice in the two groups that received higher doses of BaP (i.e., 2.5 and 6.25 mg / kg) showed a lower percentage of spontaneous alternations compared to the control mice (n = 9; *p* < 0.05), implicating that chronic exposure to the higher doses of BaP impair short-term spatial memory in mice.

### BaP exposure induced an anxiety-like behavior in mice

**Fig 2:**
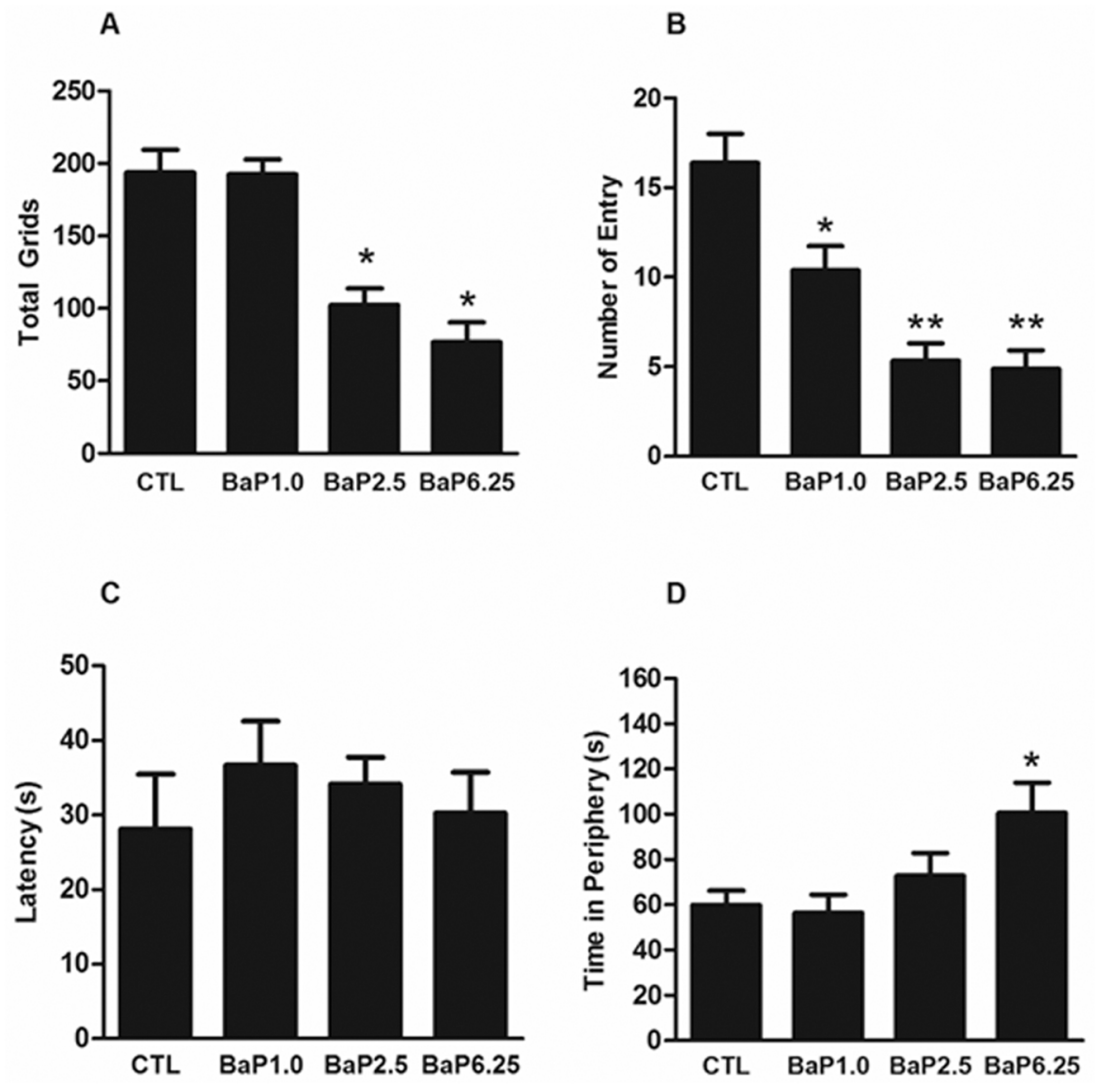
The effects of BaP on mice behavior. An open field test was used to evaluate the animal’s behavioral performance, including total grids (A), number (B) and latency (C) of entry to the central area, and the length of time in the periphery zone (D). n = 9 per BaP-treated group, n = 10 in control. Values are mean ± SEM. *, *p* < 0.05 compared to CTL.

To evaluate the impacts of BaP on exploratory locomotor activity and mood changes, open field tests were conducted after chronic exposure of the mice to BaP. Exploratory locomotor activity was assessed by the number of grids crossed by each mouse within the testing chamber, a preferred behavioral marker in exploratory behavior. The results of one-way ANOVA analysis showed a significant effect of BaP treatment on the number of crossing grids (F_(3, 35)_ = 22.78, *p* < 0.01). Mice in the two groups receiving higher BaP doses (2.5 and 6.25 mg/kg) crossed fewer grids than control mice. However, there was no such effect in the mice receiving the low BaP dose (1.0 mg/kg) (Fig 2A). Anxiety-like behavior was also assessed by determining the number of entries and the length of time spent exploring the central area of the chamber or hugging the perimeter chamber walls. BaP treated animals entered the center area with significantly less frequency than the control mice (F_(3, 35)_ = 16.98, *p* < 0.01) (Fig 2B). However, no difference was detected in the latency that occurred prior to entering the center of the arena (Fig 2C) among the 4 groups (F_(3, 35)_ = 0.449, *p* = 0.720). Only the mice exposed to 6.25 mg/kg of BaP spent more time in the periphery area (F_(3, 35)_ = 4.061, *p* <0.05) (Fig 2D). These results indicate that chronic BaP exposure induces a significant amount of anxiety-like behavior in mice.

### BaP inhibited the levels of NR2B gene expression

**Fig 3:**
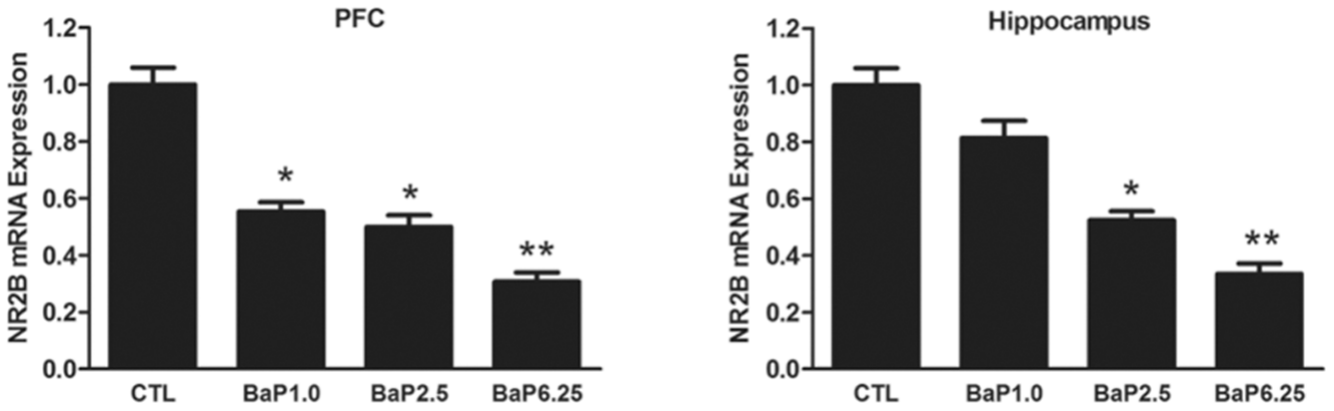
BaP exposure decreases the level of NR2B gene transcription in the PFC and hippocampus. Total RNA was isolated from the two brain regions. NR2B mRNA expression was determined by RT-qPCR, and 18S was used as an internal control. The results are presented as the average ratio vs. control ± SEM of seven independent experimental animals; *, *p* < 0.05; **, *p* < 0.01 compared with control levels.

To understand the mechanisms underlying the altered behavioral effects induced by BaP, we examined mRNA levels of the NR2B gene in the PFC and hippocampus brain regions of these mice. Normalized results are shown in Fig 3. The expression of NR2B mRNA significantly decreased in BaP-exposed mice in dose-dependent manner in both the PFC and hippocampus. In the PFC, BaP significantly inhibited NR2B expression in all three BaP-exposure groups (F_(3, 24)_ = 50.61, *p* < 0.01) while in the hippocampus, only mice in the two higher BaP dose groups (2.5 and 6.25 mg/kg) were affected (F_(3, 24)_ = 35.25, *p* < 0.01).

### BaP increased levels of DNA methylation in the NR2B promoter

**Fig 4:**
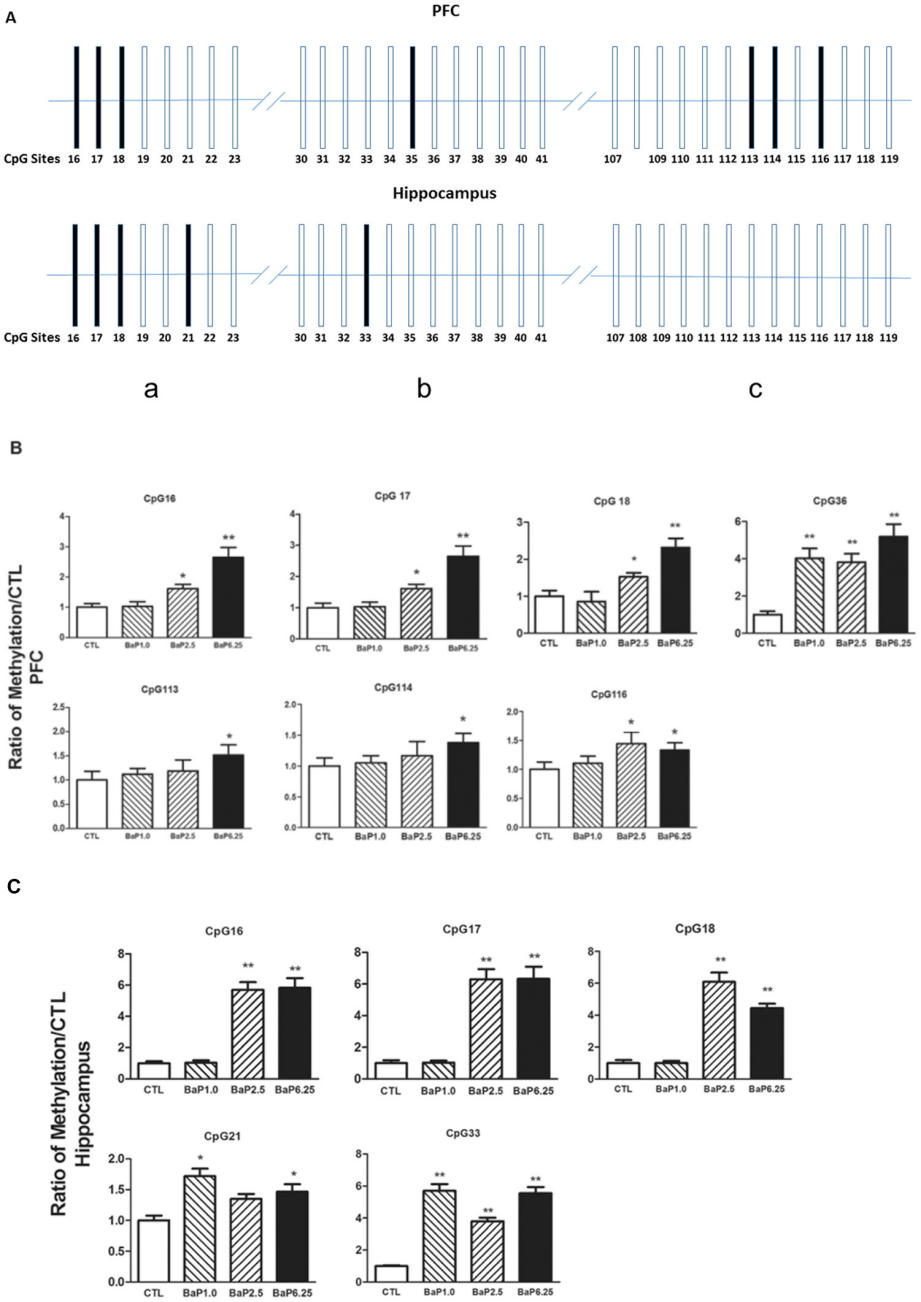
Effects of BaP on DNA methylation of the NR2B gene promoter. (A) Schematic diagram of selected CpG sites in the mouse NR2B gene promoter. Letters a-c represent the three regions in the NR2B promoter, respectively (Qiang et al., 2010). Each bar represents an individual CpG. The black bars represent a significant increase in methylation and the empty bars represent unchanged levels of methylation. The level of DNA methylation in the NR2B promoter was determined by pyrosequencing. The relative levels of NR2B methylation per CpG sites were assessed in the PFC (B) and hippocampus (C). Data are shown as the average of 5 separate experimental animals. Values are presented as the means ± SEM and represent the fold increase over the control group (control = 1). *, *p* < 0.05; **, *p* < 0.01 compared with control levels.

The potential epigenetic modifications induced by chronic exposure to BaP in the NR2B promoter were assessed. The levels of DNA methylation in 33 CpG sites in the region a, b and c selected from the NR2B promoter [33] were measured using bisulfate pyrosequencing. Of these sites, the methylation levels of CpG16, 17, 18, 35, 113, 114 and 116 in the NR2B promoter in the PFC were significantly higher in the BaP-treated mice than in the control mice. In the hippocampus, the methylation levels of CpG16, 17, 18, 21 and 33 were significantly higher in the BaP-treated mice than in the control mice (Fig 4A). The detailed changes in DNA demethylation levels in each of the individual CpG sites of the PFC (Fig 4B) and hippocampus (Fig 4C) after BaP treatment were dose-dependent.

Taken together, these data indicate that BaP-exposure induces an increase in DNA methylation in the NR2B promoter and a concomitant down-regulation of NR2B gene transcription. These alterations may contribute to the behavioral deficits observed in this study in Bap-treated mice.

## Discussion

The aim of the present study was to explore whether DNA methylation of the NR2B gene contributes to BaP-induced deficits in memory and stimulates anxiety-like behavior. To address this aim, a chronic BaP-exposure animal model was developed and behavioral tests were conducted. We observed that administration of BaP impaired short-time memory and behavioral performance. These effects were concomitant with a BaP-induced reduction in NR2B mRNA and a hypermethylation of the NR2B promoter in a dose-dependent manner. These results suggest that BaP causes brain disorders through epigenetic mechanisms associated with gene expression and neurotransmitter function. The harmful effects of exposure to BaP leading to behavioral and cognitive functional impairments were first discovered in the late 20th century [7, 38, 39, 40] and were found to affect all age groups ranging infants exposed during lactation [41] to adults [7]. However, there has been controversy regarding the doses that are likely to produce adverse effects. For example, in a sub-acute exposure in a Y-maze test, mice injected with the two lowest doses of BaP (0.02 and 0.2 mg/kg) showed a learning disability, but no effect was observed in mice treated with the higher doses (2–200 mg/kg) [42]. Another study in which a hole board test and elevated-plus maze were used, higher doses of BaP (20 and 200 mg/kg) reduced anxiety-related behavior [7]. In a separate report of chronic BaP exposure, Xia et al., found that a BaP dose of 6.25 mg/kg significantly impacted learning and memory [14]. Currently, among our chronic BaP-treated animals, mice in the higher dose groups (2.5 mg/kg and 6.25 mg/kg) showed a lower percentage of spontaneous alternations, which supports that BaP-induced effects on the CNS are dose-dependent.

There is increasing evidence that BaP affects behavioral performance, in particular by altering central neurotransmission [7, 38, 39, 42]. For example, Stephanou et al., found pollutant-induced changes in the monoaminergic system in adult rats [39] in the striatum, hypothalamus and midbrain. Another study found an increase in serotonin levels in the midbrain and cortex [38]. NMDA receptors are crucial in the CNS. These receptors are not only involved in a wide range of brain functions, including synaptic plasticity, learning and memory [43]. But they also represent a site of major vulnerability to environmental insults and risk factors for adverse effects on the brain. In particular, dysregulation of the NR2B receptor subunit has recently been associated with cognitive deficits, drug addiction and psychiatric disorders [33, 44, 45]. Polymorphisms in the NR2B are associated with cognitive disorders in schizophrenia, Alzheimer’s disease, Parkinson’s disease, obsessive-compulsive disorder, and bipolar disorder [35, 46–49]. In particular, an association between inhaled BaP and impaired NMDA-dependent LTP in rat dentate gyrus has been reported [12]. Moreover, in utero BaP exposure was found to impair the function of the somatic sensory cortex of the offspring of exposed-dams [7]. Interestingly, BaP-specific antibodies reversed the impaired behavioral performances observed in mice exposed to BaP [50]. These neurotoxic effects were further supported by evidence of a reduction in NR2B mRNA levels [29, 31]. Due to the role of NMDA receptors in learning, memory and emotional behavior [51], we examined the potential changes in the expression of the NR2B gene associated with these neurobehavioral impairments. As expected, a down-regulation of NR2B gene expression was found in the PFC and hippocampus of BaP-exposed mice, suggesting that reduced expression of NR2B may be responsible for BaP-induced neurobehavioral abnormalities. However, the exact mechanisms underlying BaP-induced neurotoxic effects through NR2B remain unknown.

Epigenetic mechanisms have been widely implicated in various aspects of CNS functions, such as synaptic plasticity [52], and have been associated with learning and memory through alterations in gene expression [53]. Specifically, alterations in DNA methylation of the NR2B gene following ethanol exposure has been previously reported to be involved in development of adverse drinking behavior [54]. The dynamic changes in DNA methylation in the promoter regions of the NR2B gene in response to ethanol insult have been reported in our earlier study [33, 34, 37]. *In vitro* and *in vivo* experiments, alterations in DNA methylation of the 5’-regulatory regions in the NR2B promoter have confirmed their regulatory role in controlling gene transcription [33]. Therefore, in the current study, we chose the same regions in the promoter of the NR2B gene, where CpG sites had been identified to mediate ethanol insult, to assess the potential alteration of DNA methylation associated to BaP-induced neurobehavioral performance. We found that the levels of DNA methylation in several CpG sites were significantly increased in both the PFC and hippocampus of BaP-exposed mice compared to the control mice. From the results in this study, it seems that the PFC is more sensitive to BaP due to the lower dosage group reduced NR2B expression; while in hippocampus the methylation level increased at a lower dosage of BaP. Therefore, further study is needed to find out which brain region is more sensitive to BaP toxic effect.

Collectively, chronic BaP exposure increases methylation levels in the NR2B gene promoter concomitantly with the down-regulation of NR2B mRNA expression. These changes may contribute to BaP-induced neurotoxic effects on behavioral performance. Therefore, the vulnerability of NR2B may represent an important molecular target for environmental toxicants.

## Abbreviations

BaP: Benzo(a)pyrene
NR2B: NMDA receptor 2B subunit
PFC: prefrontal cortex
