# Correction: Chronic Administration of Benzo(a)pyrene Induces Memory Impairment and Anxiety-Like Behavior and Increases of NR2B DNA Methylation

**DOI:** 10.1371/journal.pone.0315527

**Published:** 2024-12-05

**Authors:** Wenping Zhang, Fengjie Tian, Jinping Zheng, Senlin Li, Mei Qiang

In Materials and Methods, the BaP treatment section is not supported by a reference to a prior study in mice. Reference 35 of the section was incorrectly included to support the sentence “The mice administered with different doses of BaP by intraperitoneal [35] injections (volume 50μl) twice a week for 12 weeks.”

## References

[1] Zhang W, Tian F, Zheng J, Li S, Qiang M (2016) Chronic Administration of Benzo(a)pyrene Induces Memory Impairment and Anxiety-Like Behavior and Increases of NR2B DNA Methylation. PLoS ONE 11(2): e0149574. doi: 10.1371/journal.pone.0149574 26901155 PMC4768874

